# A Ratiometric Fluorescent Sensor Based on Silicon Quantum Dots and Silver Nanoclusters for Beef Freshness Monitoring

**DOI:** 10.3390/foods12071464

**Published:** 2023-03-29

**Authors:** Yue Sun, Xiaodong Zhai, Xiaobo Zou, Jiyong Shi, Xiaowei Huang, Zhihua Li

**Affiliations:** 1 School of Food and Biological Engineering, Jiangsu University, Zhenjiang 212013, China; 2 Institute of Modern Agriculture and Health Care Industry, Wencheng 325300, China; 3 Yixing Institute of Food and Biotechnology Co., Ltd., Yixing 214200, China; 4 International Joint Research Laboratory of Intelligent Agriculture and Agri-Products Processing, Jiangsu Education Department, Zhenjiang 212013, China

**Keywords:** fluorescent sensor, silicon quantum dots, silver nanoclusters, beef freshness, intelligent packaging

## Abstract

A ratiometric fluorescent sensor with hydrogen sulfide (H_2_S) and methanthiol (CH_3_SH) sensitivity was developed to real-time monitor beef freshness. A silicon quantum dots (SiQD) and silver nanoclusters (AgNC) complex, namely SiQD-AgNC, was used as the dual emission fluorescence materials. Due to the fluorescence resonance energy transfer (FRET) effect between SiQD and AgNC, when the fluorescence of AgNC (610 nm) was quenched by H_2_S or CH_3_SH, the fluorescence of SiQD (468 nm) recovered, resulting in an increase of the fluorescent intensity ratio (*I*_468_/*I*_610_). *I*_468_/*I*_610_ showed a linear relationship with the H_2_S concentration within the concentration range of 1.125–17 μM, with a limit of detection (LOD) value of 53.6 nM. Meanwhile, *I*_468_/*I*_610_ presented two linear relationships with the CH_3_SH concentration within the concentration range of 1.125–17 μM and 23.375–38.25 μM, respectively, with a LOD value of 56.5 nM. The SiQD-AgNC complex was coated on a polyvinylidene fluoride (PVDF) film to form a portable SiQD-AgNC/PVDF film sensor. This film showed purplish red-to-cyan color changes in response to H_2_S and CH_3_SH, with LOD values of 224 nM and 233 nM to H_2_S and CH_3_SH, respectively. When the film was used to monitor beef freshness at 4 °C, its fluorescent color gradually changed from purplish red to cyan. Hence, this study presented a new ratiometric fluorescent sensor for intelligent food packaging.

## 1. Introduction

Beef is one of the most consumed meats in the world. Cold storage (0–4 °C) is a common preservation method for fresh beef because the original nutrition, taste, and flavors could largely be maintained under this condition. However, beef spoilage inevitably occurs under cold storage due to microbial contamination and enzymatic reaction, which leads to food waste issues and food safety risk [1]. To date, most methods to evaluate beef freshness, such as the determination of total volatile basic nitrogen (TVBN), thiobarbituric acid reactive substances, and microbial population, are generally time-consuming. As a result, these methods can hardly determine real-time beef freshness to meet the requirements of both manufacturers, consumers, and supervisors. Hence, it is always highly desirable to develop novel methods to evaluate real-time beef freshness.

Intelligent packaging has received great interest in the recent two decades. Intelligent packaging was defined as a packaging system that is capable of carrying out intelligent functions, such as detecting, sensing, recording, tracing, communicating, and applying scientific logic, to facilitate decision making to extend shelf life, enhance safety, improve quality, provide information, and warn about possible problems [2]. Among various intelligent packaging systems, food freshness indicators are extremely attractive. Nowadays, freshness indicators for meats, fish, vegetables, fruits, and so forth have been widely reported [3,4]. Generally, the principle of freshness indicators was that they were sensitive to volatile gases produced from foods and able to show detectable signal changes, such as color [5] and electrical changes [6].

During beef spoilage, volatile sulfides, including hydrogen sulfide, mercaptans, and thioethers, are generated due to the decomposition of proteins [7]. Hence, these sulfides have been regarded as one of the important compounds to evaluate beef freshness. Nowadays, the most common sulfides sensors are electrochemical sensors [8]. However, electrochemical sensors can hardly be integrated with an intelligent packaging system due to the requirement of power sources. By contrast, colorimetric sensors are more simple, portable, and easy to fabricate. To date, there are still limited reports on developing volatile sulfides-responsive colorimetric sensors for beef freshness monitoring [9,10,11,12,13]. Therefore, more novel colorimetric sensors are highly desirable.

Fluorescent sensors have been widely applied in many fields, including biology, physiology, medicine, and pharmacology, by virtue of their high sensitivity, fast response time, and so on. In recent years, fluorescent sensors have also been reported to monitor meat freshness or spoilage in intelligent packaging [14,15,16]. As to the beef freshness, Long, Cao, Jin, Yuan, Han, and Wang [9] developed a fluorescent probe of H_2_S, and its blue fluorescent intensity increased with the increase of H_2_S concentration. When this probe was used to monitor beef freshness, its blue color gradually became deeper with storage time. However, one shortage of such fluorescent sensors was that the changes of single fluorescent intensity generally were not highly visible for naked eyes. By contrast, ratiometric fluorescent sensors with diverse color changes are generally easier to recognize by naked eyes. Meanwhile, ratiometric fluorescent sensors are highly anti-jamming compared to single-emission fluorescent sensors because ratiometric fluorescent sensors could provide built-in self-calibration by calculating the strength ratio of the two fluorescent signals, so as to provide more accurate quantification [17]. Recently, we developed a ratiometric fluorescent sensor based on carbon dots-copper nanoclusters with H_2_S sensitivity to monitor chilled pork and chicken spoilage [18]. This sensor showed red-to-blue color changes with the storage of these meat samples. Nevertheless, to the best of our knowledge, studies on developing ratiometric fluorescent sensors with volatile sulfides sensitivity to monitor beef freshness have not been reported yet.

Hence, in this study, we aimed to develop a new ratiometric fluorescent sensor to monitor beef freshness based on its sensitivity to volatile sulfides. The sensor was composed of silicon quantum dots (SiQD) and silver nanoclusters (AgNC). The SiQD and AgNC were combined through dehydration condensation reaction to form a SiQD-AgNC complex. As a result, the fluorescence resonance energy transfer (FRET) effect was formed between SiQD and AgNC, namely the emission light of SiQD served as the excitation light of AgNC. When the SiQD-AgNC complex was exposed to volatile sulfides, the red fluorescent emission of AgNC was quenched due to the production of the Ag-S bond, while the cyan fluorescent emission of SiQD recovered, inducing a red-to-cyan color change. In the application experiment, the SiQD-AgNC complex was coated onto a polyvinylidene fluoride (PVDF) film to form a portable sensor to detect volatile sulfides and monitor beef freshness.

## 2. Materials and Methods

### 2.1. Materials

Fresh beef was purchased from the local market (Zhenjiang, China). Silver nitrate, polymethacrylic acid sodium salt (PMAA, Mw = 9500), 3-aminopropyltriethoxysilane (APTES), *D*-glucose, 1-ethyl-3-(3-(dimethylamino) propyl)-carbodii-mide (EDC), and N-hydroxysulfosuccinimide (NHS) were purchased from Singapore. A high-pressure mercury lamp (λ = 365 nm, 100 W) was purchased from Sylvania (Lucas, OH, USA).

### 2.2. Synthesis of AgNC

AgNC was synthesized by using the one-step illumination method according to a previous study with a slight modification [19]. First, 48 mL of 0.05 M silver nitrate solution and 6 mL of PMAA solution were mixed for 10 min under dark, and then kept for 20 min. After that, the mixture was transferred to a beaker (1 L) and illuminated with the high-pressure mercury lamp for 15 min to synthesize AgNC. Finally, the obtained AgNC solution was dialyzed for 24 h using a 500 Da dialysis tube, and then freeze-dried for further use.

### 2.3. Synthesis of SiQD

SiQD was synthesized by using the one-step reducing method according to a previous study with a slight modification [20]. Firstly, 4.5 g of *D*-glucose was dissolved in 40 mL of water. Then, 5 mL of APTES was dropwise added to the *D*-glucose solution under stirring and kept stirring for 48 h under 25 °C. During this process, the mixture gradually changed from colorless to brown, indicating the formation of SiQD. Finally, the obtained SiQD solution was dialyzed for 24 h using a 500 Da dialysis tube, and then freeze-dried for further use.

### 2.4. Synthesis of SiQD-AgNC Complex

Firstly, 250 μL of mixture containing 10 mg/mL of EDC and 10 mg/mL of NHS was added to 3 mL of AgNC solution (1 mg/mL) and then stirred for 2 h. Then, 500 μL of SiQD solution (0.2 mg/mL) was added to the above mixture and stirred then for 3 h to form the SiQD-AgNC complex. Finally, the obtained SiQD-AgNC solution was dialyzed for 24 h using a 1000 Da dialysis tube, and then freeze-dried for further use.

### 2.5. Detection of H_2_S and CH_3_SH

Firstly, H_2_S was generated from the reaction between HNO_3_ and Na_2_S. Here, the molarity of nitric acid was three times the molarity of sodium sulfide to ensure that the molecular number of H_2_S was equal to the molecular number of Na_2_S. Then, H_2_S was blown to the SiQD-AgNC solution using N_2_, and the fluorescent spectra of the SiQD-AgNC solution were recorded. Similarly, CH_3_SH was generated from the reaction between HNO_3_ and CH_3_NaS, and then reacted with SiQD-AgNC solution.

### 2.6. Volatile Compounds Analysis of Beef Samples

The determination of volatile compounds of beef samples was conducted by using a gas chromatography-mass spectrometer (GC-MS) combined with the solid-phase microextraction (SPME) method, according to our previous study [10]. Briefly, 6 g of beef samples were put into a headspace vial (15 mL). This vial was then sealed with a silicone septum and screw-thread cap, and equilibrated for 15 min at 60 °C. The volatile gases from beef samples were firstly extracted for 40 min at 60 °C using an SPME fiber assembly (50/30 μm DVB/CAR/PDMS, ANPEL Laboratory Technologies Inc. (Shanghai, China)). After extraction, the volatile gases were desorbed into the GC injector at 250 °C for 5 min with a splitless mode on a Trace Ultra ITQ1100 GC-MS system (Thermo Scientific, Waltham, MA, USA). In this system, the volatile gases were separated using a DB-WAX column (60 m × 0.25 mm × 0.25 μm; Agilent Technologies, Santa Clara, CA, USA) with a helium flow rate of 1.6 mL/min. The temperature was set at 40 °C for 4 min, then a ramp of 5 °C/min until 100 °C, followed by a ramp of 6 °C/min to 220 °C, and finally at 220 °C for 3 min. MS detection was performed with a source temperature of 230 °C, quadrupole temperature of 200 °C, electron energy of −70 eV, and the mass scan range of *m*/*z* 33–450.

### 2.7. Application of Sensor in Monitoring Beef Freshness

Firstly, 50 μL of SiQD-AgNC solution was added onto a PVDF film, and then dried in an oven at 60 °C for 20 min. The obtained film was expressed as the SiQD-AgNC/PVDF film. The SiQD-AgNC/PVDF film was adhered in the internal surface of the lid of a polyethylene terephthalate (PET) box which contained a whole piece of 150 g fresh beef. The PET box was stored in the dark in a refrigerator with 4 °C. The fluorescent photograph of the PET box was obtained by using a dark-box UV analyzer (CBIO-UV6, Saibaiao Technology Co., Ltd., Beijing, China).

The total viable counts (TVC) of beef were measured using the plate count method following the Chinese standard GB 4789.2–2016. Briefly, 25 g of beef sample was homogenized with 225 mL of phosphate buffer solution. Then, the homogeneous solution was filtered, and the supernate was serially diluted at a volume ratio of 10. After that, 1 mL of the diluted solution was carefully spread on bacteria-counting agar plates. These procedures were carried out in a sterile environment. Finally, agar plates were incubated at 35 °C with different times, and the number of colonies was recorded. The bacterial counts were expressed as colony-forming units (CFU) per gram of beef, and then transformed to base 10 logarithm values, namely log_10_(CFU/g) or lg(CFU/g) [21].

## 3. Results and Discussions

### 3.1. Optimization of SiQD and AgNC Synthesis

First, SiQD was synthesized at room temperature using *D*-glucose as a reducing agent and APTES as a silicon source. Due to the presence of the amino group at the end of APTES, the surface of SiQD contained many amino groups, which prevented agglomeration of SiQD by charge repulsion. At the same time, AgNC was synthesized by photoreduction using silver nitrate as a silver source and PMAA as a protective agent. Due to the presence of PMAA, the surface of AgNC contained numerous carboxyl groups, which prevented AgNC agglomeration by charge repulsion.

**Figure 1:**
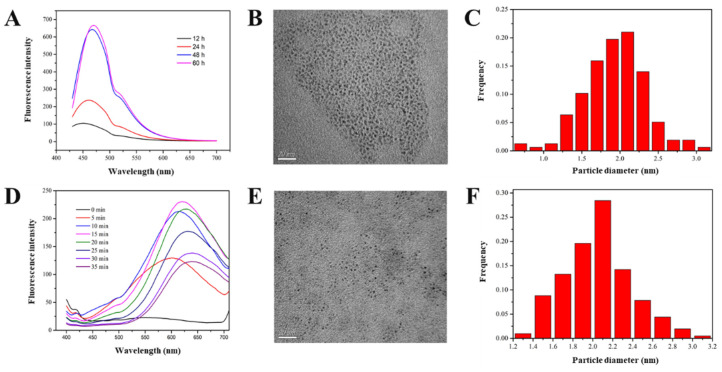
(**A**) The fluorescent spectrum of SiQD with different synthesis time. (**B**) TEM of SiQD (scale bar 20 nm). (**C**) Size distribution of SiQD. (**D**) The fluorescent spectrum of AgNC with different synthesis time. (**E**) TEM of AgNC (scale bar 20 nm). (**F**) Size distribution of AgNC.

The synthesis time was an important parameter for both SiQD and AgNC. As shown in Figure 1A, under a 400 nm excitation light, the fluorescent intensity of SiQD increased in the first 48 h, and remained nearly unchanged, even the synthesis time was extended to 60 h. This was because APTES was first reduced to form a small nucleus, and these small nuclei with high specific surface areas were unstable. Subsequently, larger and more stable nanocrystals were formed through the Ostwald maturation process [22]. Once the ripening process was completed, extending the reaction time would not lead to the increase of particle size and fluorescent intensity. Hence, 48 h was the optimal synthesis time for SiQD.

The change of fluorescent intensity of AgNC is shown in Figure 1D. Under a 500 nm excitation light, the fluorescent intensity firstly increased with time in the initial 15 min. However, when the illumination time was longer than 15 min, the fluorescent intensity decreased and the fluorescent peak gradually red shifted. This indicated that the particle size of AgNC increased with illumination time. As the low-density electronic states of the smaller nanoclusters are the main factor for their fluorescence generation, the increase of AgNC particle size led to the decrease of their fluorescence. Therefore, 15 min was selected as the optimal synthesis time.

TEM images of SiQD and AgNC particles are shown in Figure 1B,E, respectively. It can be seen that both SiQD and AgNC showed a dispersive state without obvious aggregation. According to TEM images, their particle sizes were calculated and size distributions are shown in Figure 1C,F, respectively. The average particle sizes of SiQD and AgNC were 2.14 and 1.99 nm, respectively.

### 3.2. Fluorescence Characteristics of SiQD and AgNC

**Figure 2:**
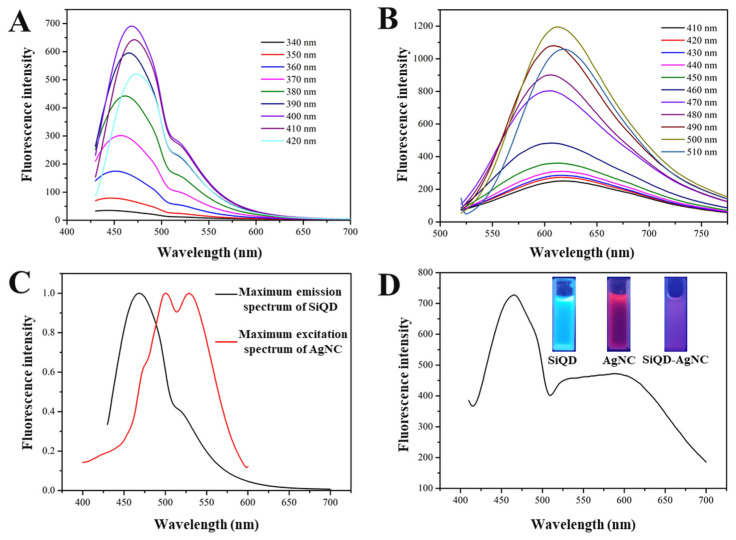
(**A**) The emission spectra of SiQD at different excitation light. (**B**) The emission spectra of AgNC at different excitation light. (**C**) The maximum emission spectrum of SiQD and the maximum excitation spectrum of AgNC. (**D**) The emission spectrum of the SiQD-AgNC complex, and the fluorescent photos (insets) of the SiQD, AgNC, and SiQD-AgNC complex.

Figure 2A shows the fluorescent emission spectra of SiQD at different excitation wavelengths. When the excitation wavelength increased from 340 to 400 nm, the intensity of the emission spectrum gradually increased, while the maximum emission wavelength shifted slowly from 450 to 468 nm. When the excitation wavelength was greater than 400 nm, the intensity of the emission peak decreased. Therefore, the maximum excitation wavelength of SiQD was 400 nm, and the corresponding emission wavelength was 468 nm.

Figure 2B shows the fluorescent emission spectra of AgNC at different excitation wavelengths. When the excitation wavelength increased from 410 to 500 nm, the intensity of the emission spectrum gradually increased, and the wavelength of the maximum emission peak remained almost constant. When the excitation wavelength was 510 nm, the intensity of the emission peak decreased. Therefore, the maximum excitation wavelength of AgNC was 500 nm, and the corresponding emission wavelength was 610 nm.

The maximum emission spectrum of SiQD and the maximum excitation spectrum of AgNC are shown in Figure 2C. It can be seen that there was a large overlap between these two peaks within 450–550 nm, which made the FRET between SiQD and AgNC possible. Figure 2D shows the fluorescent emission spectrum of SiQD-AgNC complex at 400 nm excitation light. It can be seen that the SiQD-AgNC composite simultaneously showed the emission peaks of SiQD at 468 nm and AgNC at 610 nm. Fluorescent photographs of SiQD, AgNC, and SiQD-AgNC complexes are shown in Figure 2D. At the excitation light of 365 nm, SiQD and AgNC showed cyan and red color, respectively, while SiQD-AgNC showed purplish red, namely the hybrid color of cyan and red. These results indicated that a dual emission fluorescent sensor was successfully developed.

### 3.3. Principle of Detection

**Figure 3:**
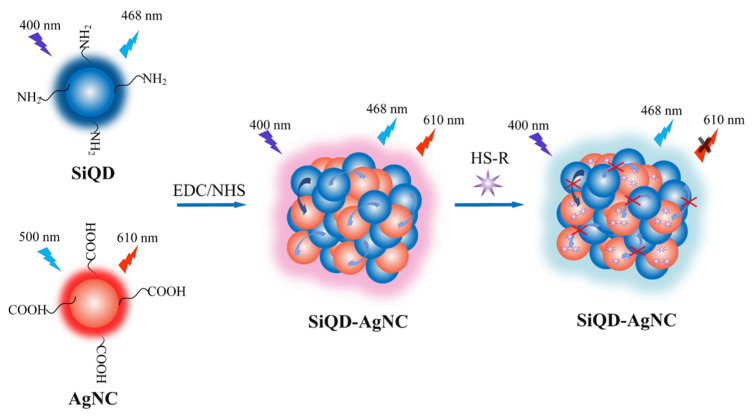
The detection principle of the SiQDs-AgNCs complex to hydrogen sulfide and mercaptan.

In this study, the developed AgNC-SiQD complex was supposed to be sensitive to H_2_S and mercaptan. The detection principle of the developed fluorescent sensor to H_2_S and mercaptan is shown in Figure 3. During the synthesis of the AgNC-SiQD complex, the positive amino group on the surface of SiQD and the negative carboxyl group on the surface of AgNC could form amido bonds, with EDC/NHS as an activator. As a result, when the distance between SiQD and AgNC was equal to or less than 10 nm, the emission light of SiQD could act as the excitation light of AgNC, which was regarded as the FRET effect.

It is generally known that the Ag atom has a low empty d electron orbital. When the electron pair of the S atom enters the empty *d*-electron orbital of the Ag atom, it is easy to form the Ag-S metal-ligand bond to cause fluorescence quenching of AgNC. As a result, the emission light of SiQD could be enhanced because AgNC no longer absorbed the emission light of SiQD.

The fluorescence quenching of AgNC in response to H_2_S was due to the formation of Ag_2_S that had no fluorescence characteristics. However, the mechanism of fluorescence quenching caused by the combination of mercaptan and AgNC was still unclear. According to previous literature, the fluorescence quenching of AgNC seems to be related to the change of particle size and surface protectant. For example, Zhang, et al. [23] prepared AgNC with polyethyleneimine (PEI) as the protecting agent. When substances containing mercaptan (cysteine, homocysteine, and glutathione) were added to AgNC, mercaptan could combine with AgNC to destroy the binding site between PEI and AgNC. This would reduce the positive surface charge of AgNC, thus causing aggregation of AgNC under Van der Waals gravity to form large particle aggregates without fluorescence characteristics. Li and Wei [24] prepared AgNC with DNA as the protecting agent. When mercaptan-containing substances (cysteine and glutathione) were added to AgNC, the polarity of the solution changed. Meanwhile, the formation of the Ag-S bond changed the secondary structure of DNA. The fluorescence lifetime experiment showed that the energy transfer from the AgNC donor to the mercaptan receptor occurred. These factors together led to the fluorescence quenching of AgNC. In this experiment, the fluorescence quenching of AgNC induced by mercaptan was probably due to the formation of the Ag-S bond that destroyed the cross-linking between AgNC and PMAA, causing the aggregation of AgNC.

### 3.4. Optimization of pH and Ionic Strength

**Figure 4:**
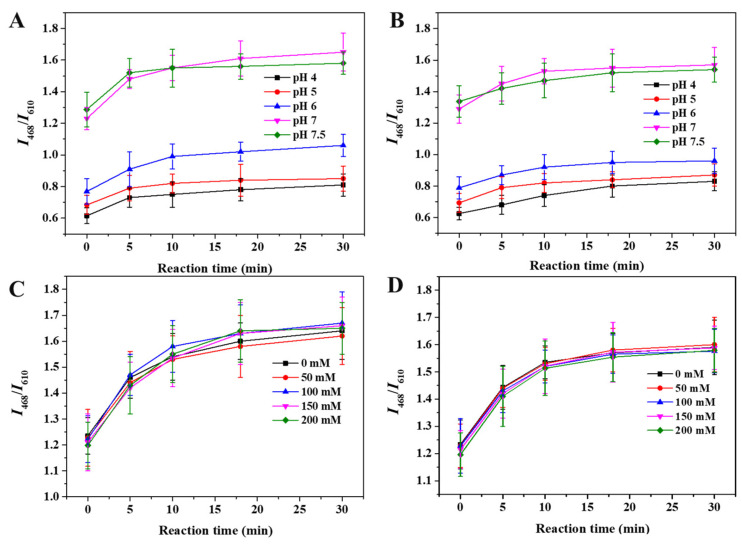
The changes of *I*_468_/*I*_610_ of SiQD-AgNC reacting with (**A**) H_2_S and (**B**) CH_3_SH under different pH. The changes of *I*_468_/*I*_610_ of SiQD-AgNC reacting with (**C**) H_2_S and (**D**) CH_3_SH under different ion strength.

The reaction of SiQD-AgNC with H_2_S and CH_3_SH was greatly affected by pH. As shown in Figure 4A,B, the initial fluorescent intensity of SiQD-AgNC was different at different pH. Between pH 4 and 7.5, the ratio of fluorescent intensity *I*_468_ of SiQD and fluorescent intensity *I*_610_ of AgNC, namely *I*_468_/*I*_610_, gradually increased with the increase of pH. Especially, when pH increased from 6 to 7, SiQD-AgNC increased greatly. When the pH was less than 4 or more than 7.5, the solution became very unstable, and so the reaction of SiQD-AgNC with H_2_S and CH_3_SH was measured only within pH 4–7.5. At different pH values, the reactions tended to reach equilibrium within 30 min. At pH 7, the change of *I*_468_/*I*_610_ before and after the reaction was the largest. The *I*_468_/*I*_610_ value increased 0.42 in response to H_2_S (Figure 4A) and increased 0.28 in response to CH_3_SH. Therefore, the SiQD-AgNC solution was adjusted to pH 7 for further study.

At pH 7, different concentrations of sodium nitrate were added to the solution to determine the effect of ionic strengths on the reaction between SiQD-AgNC and H_2_S or CH_3_SH. As shown in Figure 4C,D, the initial *I*_468_/*I*_610_ value of the solution decreased only from 1.23 to 1.20 when the concentration of sodium nitrate increased from 0 to 200 mM. Hence, the ionic strength had no significant effect on the stability of the solution in the range of 0–200 mM. After adding 17 μM H_2_S (Figure 4C) or CH_3_SH (Figure 4D) to solutions with different ionic strengths, the changes of *I*_468_/*I*_610_ values over time were also very close, indicating that ionic strength has no significant influence on the reaction.

### 3.5. Sensitivity of SiQD-AgNC to H_2_S and CH_3_SH

**Figure 5:**
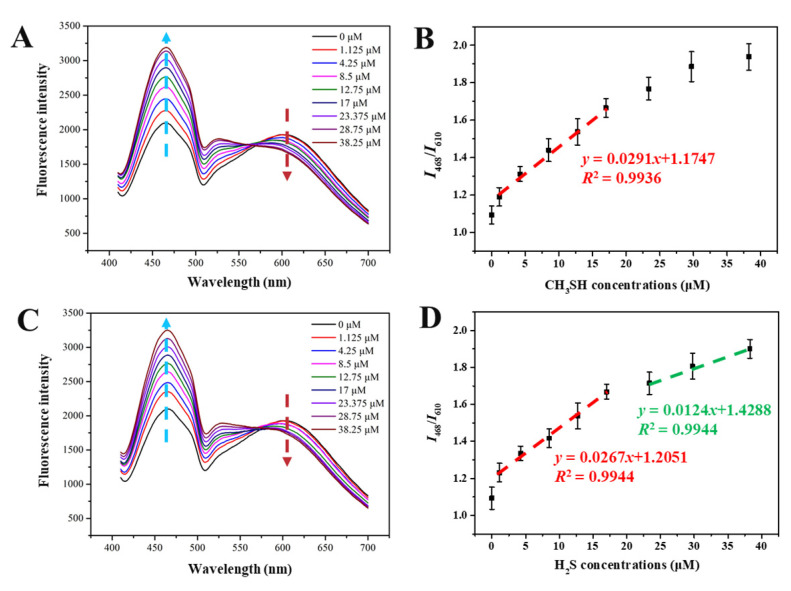
The fluorescent spectra of SiQD-AgNC reacting with different concentration of (**A**) H_2_S and (**C**) CH_3_SH. The relation between *I*_468_/*I*_610_ and the concentrations of (**B**) H_2_S and (**D**) CH_3_SH.

The sensitivity of SiQD-AgNC was investigated by reacting with different concentrations of H_2_S and CH_3_SH. As shown in Figure 5A, with the increase of H_2_S concentration, the fluorescent intensity of SiQD-AgNC at 610 nm decreased, while fluorescent intensity at 468 nm increased. Here, the fluorescent intensity at 468 nm changed more significantly than that at 610 nm, which may be because the spectrum of SiQD overlapped with the spectrum of AgNC at 610 nm, weakening the decline of the overall spectrum at 610 nm. The relationship between *I*_468_/*I*_610_ value and H_2_S concentration is shown in Figure 5B. With the increase of H_2_S concentration, the *I*_468_/*I*_610_ value gradually increased. There was a linear relationship between the value of *I*_468_/*I*_610_ and H_2_S concentration within the concentration range of 1.125–17 μM, and the *R*^2^ of the calibration curve was 0.9936. Similarly, as shown in Figure 5D, there were linear relationships between the *I*_468_/*I*_610_ value and CH_3_SH concentration, in the CH_3_SH concentration range of 1.125–17 μM and 23.375–38.25 μM, respectively. The *R*^2^ of the calibration curves was both 0.9944. According to these calibration curves, the limit of detection (LOD) values of SiQD-AgNC to H_2_S and CH_3_SH were 53.6 nM and 56.5 nM, respectively, using the following Equation (1):

LOD = 3 *K*/*N* (1)

where *K* is the standard deviation of blank measurements for 13 times, and *N* is the slope of the linear calibration curve.

The linear detection range and LOD of SiQD-AgNC to H_2_S were compared with reported fluorescent sensors. As shown in Table 1, the developed SiQD-AgNC in this work was comparable to other sensors based on dyes, quantum dots, and nanoclusters.

### 3.6. Selectivity of SiQD-AgNC to H_2_S and CH_3_SH

**Figure 6:**
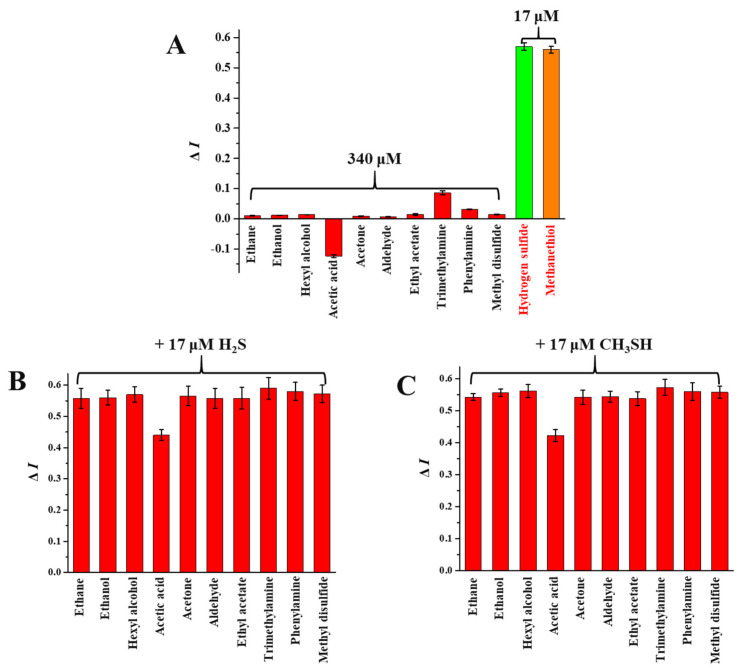
(**A**) The Δ*I* values of SiQD-AgNC reacting with 340 μM of other volatile gases or 17 μM of H_2_S and CH_3_SH.(**B**) The Δ*I* values of SiQD-AgNC simultaneously reacting with 340 μM of other volatile gases in the presence of 17 μM of H_2_S. (**C**) The Δ*I* values of SiQD-AgNC simultaneously reacting with 340 μM of other volatile gases in the presence of 17 μM of CH_3_SH.

To verify the selectivity of SiQD-AgNC to H_2_S and CH_3_SH, the volatile gases of beef during storage at 4 °C was firstly determined. As shown in Table 2, 33 volatile gases were identified in total, mainly including alkanes, alcohols, aldehydes, esters, amines, and carbon dioxide. These compounds were very close to previous studies [32,33]. Generally, alkanes are mainly generated from the cracking of alkoxy in fatty acids, and alcohols result from the free-radical promoted saccharide decomposition due to lipid oxidation. Aldehydes, ketones, and acids are mainly the result from the oxidative degradation of fats [33]. Amines and sulfur-containing substances are generally generated from the decomposition of amino acids and proteins. In this study, sulfur-containing substances, including H_2_S, CH_3_SH, dimethyl disulfide, and dimethyl trisulfide, have been identified. H_2_S and CH_3_SH were detected on the 4th and 3th day, respectively. Since it is difficult to investigate the effect of each volatile compound on the selectivity of SiQD-AgNC one by one, as a compromise, we selected some representative components from their homologues or structural analogues, including hexane, ethanol, n-hexyl alcohol, acetic acid, acetone, acetaldehyde, ethyl acetate, trimethylamine, aniline, and dimethyl disulfide. As shown in Figure 6A, when the concentration of these volatile substances, except acetic acid, was 20 times the concentration of H_2_S and CH_3_SH, the *I*_468_/*I*_610_ values of SiQD-AgNC did not show obvious change (Δ*I* < 0.1). Here, acetic acid led to a decrease of *I*_468_/*I*_610_ more than 0.1, which may be because acetic acid could reduce the fluorescence of SiQD through binding to the amino group of SiQD. At the same time, these substances were mixed with H_2_S and CH_3_SH, and then these mixtures were added to the SiQD-AgNC solution to investigate their effects on Δ*I* under coexistence conditions. As shown in Figure 6B,C, SiQD-AgNC still had good significant response to H_2_S and CH_3_SH in the presence of these substances. Therefore, SiQD-AgNC could be used as a highly selective sensor for H_2_S and CH_3_SH.

### 3.7. Sensitivity of Fluorescent Film to H_2_S and CH_3_SH

**Figure 7:**
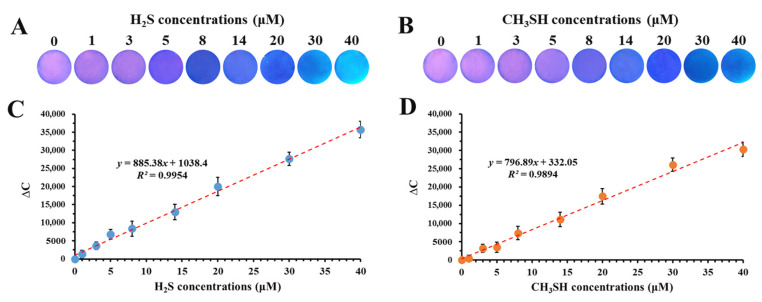
The fluorescence change of the SiQD-AgNC/PVDF film in response to (**A**) H_2_S and (**B**) CH_3_SH. The relation between ∆*C* of SiQD-AgNC/PVDF film and the concentrations of (**C**) H_2_S and (**D**) CH_3_SH.

Considering that a solid film is generally more convenient than liquid solution for use in practical food packaging, in this work, the SiQD-AgNC solution was coated onto a PVDF film to form a portable fluorescent film, named the SiQD-AgNC/PVDF film. The fluorescence changes of the SiQD-AgNC/PVDF film in response to H_2_S and CH_3_SH are shown in Figure 7A,B, respectively. Its color gradually changed from purplish red to blue and final cyan, with the increase of H_2_S or CH_3_SH concentration. To better determine the relation between fluorescent color of the SiQD-AgNC/PVDF film and gas concentration, the color change (∆*C*) of the film was calculated by using the following Equation (2):

(2) $$\Delta C={\left(R-R_{0}\right)}^{2}+{\left(G-G_{0}\right)}^{2}+{\left(B-B_{0}\right)}^{2}$$

where *R_0_, G_0_*_,_ and *B_0_* are respectively the red, green, and blue color of the film before reacting with H_2_S or CH_3_SH, while *R, G*, and *B* are respectively the red, green, and blue color of the film after reacting with H_2_S or CH_3_SH, under a 365 nm UV light.

It can be seen from Figure 7C,D that the ∆*C* increased with the increase of H_2_S and CH_3_SH concentration. A linear relation was obtained at the range of 0–40 μM for both H_2_S and CH_3_SH, with *R^2^* of 0.9954 and 0.9894, respectively. Accordingly, the limit of detection (LOD) values for H_2_S and CH_3_SH were 224 nM and 233 nM, respectively, using Equation (1).

It was also needed to mention that the reactions between the SiQD-AgNC/PVDF film and H_2_S or CH_3_SH were not reversible, which was beneficial to monitoring the real-time beef freshness.

### 3.8. Application of Fluorescent Film in Monitoring Beef Freshness

**Figure 8:**
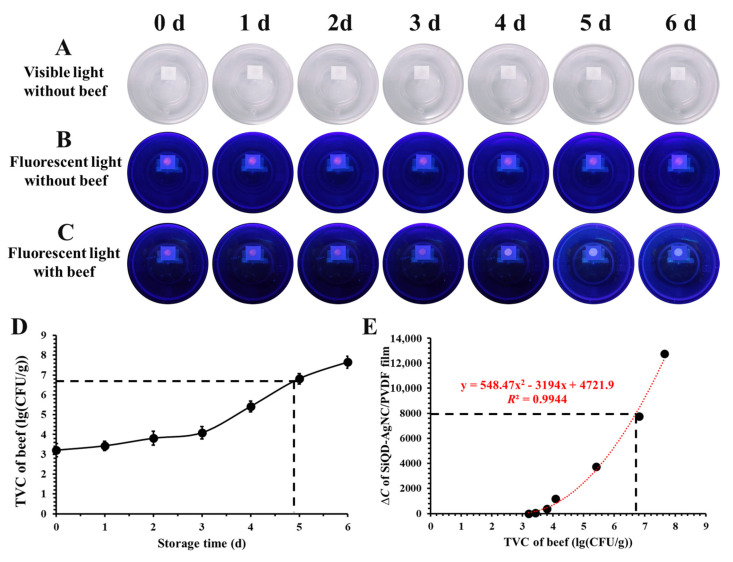
(**A**) The visible light photos and (**B**) fluorescent light photos of a polypropylene packaging box integrated with a SiQD-AgNC/PVDF film, during storage at 4 °C. (**C**) The fluorescent light photos of the packaging system with beef sample, during storage at 4 °C. (**D**) The change of TVC values of beef during storage at 4 °C. (**E**) The relation between **∆*C*** of SiQD-AgNC/PVDF film and TVC value of beef.

The SiQD-AgNC/PVDF film was used to real-time monitor beef freshness. As shown in Figure 8A, the film was adhered onto the internal surface of the lid of a transparent polypropylene packaging box. The color of SiQD-AgNC on the PVDF film was very weak, as shown in visible light photos, and this light purple color remained almost unchanged during storage (Figure 8A), indicating its good stability. This good stability could also be seen from the fluorescent light photos. As shown in Figure 8B, the purplish red color of the SiQD-AgNC/PVDF film was constant. In comparison, when the SiQD-AgNC/PVDF film was used to monitor beef freshness, its fluorescent color obviously changed from purplish red to cyan (Figure 8C). The *R*, *G*, *B* values of the SiQD-AgNC/PVDF film are shown in Table 3. It can be seen that the *R* value gradually decreased, while *G* and *B* values gradually increased, verifying its purplish red-to-cyan color change.

The freshness of beef was evaluated according to its TVC value. As shown in Table 3, the TVC of beef increased from initial 2.01 lg(CFU/g) to 7.95 lg(CFU/g) after six days. According to European legislation (EC Regulation 1441/2007, 2007), ~6.7 lg(CFU/g) is the maximum acceptable limit of TVC for raw meats [10]. In this study, the TVC of beef reached 6.7 lg(CFU/g) after nearly 4.8 day of storage (Figure 8D), indicating that the beef was inedible after 4.8 days of storage at 4 °C.

The relation between the color of SiQD-AgNC/PVDF film and the TVC value of beef is shown in Figure 8D. There was a polynomial relation between ∆*C* and the TVC value, with *R*^2^ of 0.9944. According to this polynomial relation, when the TVC value was 6.7, ∆*C* was calculated to be 7943. This indicated that if the ∆*C* value of the SiQD-AgNC/PVDF film was higher than 7943, the beef could not be consumed. Hence, the developed SiQD-AgNC/PVDF film was able to real-time monitor beef freshness for intelligent food packaging.

## 4. Conclusions

A ratiometric fluorescent sensor based on SiQD-AgNC was successfully developed. SiQD-AgNC exhibited two emission peaks at 468 and 610 nm. When SiQD-AgNC reacted with H_2_S and CH_3_SH, the fluorescent intensity of AgNC at 610 nm decreased while the fluorescent intensity of SiQD at 468 nm increased, forming a ratiometric fluorescent sensor. The optimal condition of SiQD-AgNC for H_2_S and CH_3_SH sensing was pH 7.0. Under pH 7.0, the LOD values of SiQD-AgNC were 53.6 nM and 56.5 nM for H_2_S and CH_3_SH, respectively. SiQD-AgNC showed good selectivity to H_2_S and CH_3_SH in the presence of other volatile gases generated from beef during storage. When the SiQD-AgNC/PVDF film was used to monitor beef freshness, it showed a purplish red-to-cyan fluorescent color change, and this color change was closely related to the TVC value of beef. Hence, the developed ratiometric fluorescent sensor had great potential for practical application in intelligent food packaging.

## Figures and Tables

**Table 1:** Comparison of previously reported fluorescent sensors and SiQD-AgNC in this work.

Sensor Materials	Detection Target	Linear Range (μM)	LOD (nM)	References
Isophorone-xanthene dye	H_2_S	1.0–40.0	250	[25]
6-(2, 4-dinitrophenoxy)-2-naphthonitrile	H_2_S	0–70	76	[26]
7-amino-4-methylcoumarin and fluorescein	H_2_S	0–140	31	[27]
CsPbBr3 quantum dots	H_2_S	0–100	180	[28]
Carbon quantum dots	S^2−^	0–3	62.7	[18]
Carbon quantum dots -PNBD	H_2_S	0–35	57	[29]
Gold nanoclusters	H_2_S	0.002–120	1.8	[30]
Carbon dots-copper nanoclusters	H_2_S	26–128	4.3	[31]
Silicon quantum dots- silver nanoclusters	H_2_S	1.125–17	53.6	This work

**Table 2:** The volatile compounds of beef during storage at 4 °C.

Category	Name	Relative Contents (*w*/*w*%)						
		0 d	1 d	2 d	3 d	4 d	5 d	6 d
Sulfides	Dimethyl disulfide	-	-	0.45 ± 0.22	-	0.58 ± 0.22	0.64 ± 0.35	-
	Hydrogen sulfide	-	-	-	-	0.23 ± 0.10	1.17 ± 0.61	1.68 ± 0.24
	Methanethiol	-	-	-	0.35 ± 0.23	2.48 ± 0.43	4.46 ± 1.29	3.59 ± 2.37
	Dimethyl trisulfide	-	-	-	-	-	0.74 ± 0.32	0.81 ± 0.39
Hydrocarbon	Pentane	4.17 ± 1.85	6.33 ± 2.42	3.28 ± 2.06	2.30 ± 0.15	-	4.3 0 ± 2.74	-
	Hexane	2.66 ± 0.56	5.54 ± 1.69	3.51 ± 1.78	1.57 ± 0.56	-	0.34 ± 0.24	3.16 ± 0.88
	Pentadecane	3.39 ± 0.45	1.22 ± 0.62	-	3.53 ± 0.75	1.01 ± 0.14	-	-
	2,6,10-trimethyl-dodecane	4.87 ± 0.85	-	2.10 ± 0.43	-	-	0.77 ± 0.17	-
	Heptadecane	2.40 ± 0.33	5.04 ± 0.54		4.21 ± 2.16	-	-	1.20 ± 0.53
Alcohols	1-pentanol	3.34 ± 1.84	5.08 ± 2.42	3.14 ± 1.92	2.12 ± 0.67	1.56 ± 0.33	0.81 ± 0.81	4.21 ± 0.47
	1-hexen-3-ol	2.89 ± 0.15	-	4.22 ± 0.43	3.78 ± 0.85	-	5.21 ± 1.64	-
	Ethanol	0.63 ± 0.35	1.26 ± 0.27	-	0.85 ± 0.11	-	1.53 ± 0.38	1.69 ± 0.56
	1-octene-3-ol	-	2.2 ± 0.75	2.31 ± 0.64	1.89 ± 0.76	1.45 ± 0.63	0.80 ± 0.24	-
	4-methyl-1-amyl alcohol	-	1.88 ± 0.53	-	-	-	3.22 ± 0.68	-
	Hexyl alcohol	-	-	1.57 ± 0.45	1.56 ± 0.16	1.91 ± 0.37	1.81 ± 0.55	3.56 ± 0.49
	Butanol		-	-	0.38 ± 0.12	-	3.49 ± 0.26	-
Aldehydes/ketones	Hexanal	30.59 ± 7.12	23.40 ± 7.33	20.76 ± 5.68	21.72 ± 8.24	11.88 ± 5.15	7.01 ± 2.19	24.43 ± 8.32
	Valeraldehyde	-	0.47 ± 0.14	-	0.94 ± 0.18	1.89 ± 0.15	-	-
	3-methylbutyral	-	0.56 ± 0.19	-	-	-	1.47 ± 0.13	-
	Heptanal	-	-	1.88 ± 0.37	-	-	1.08 ± 0.36	2.37 ± 0.54
	Acetone	18.44 ± 6.52	10.69 ± 5.17	5.53 ± 2.63	3.15 ± 0.35	3.25 ±1.72	2.62 ± 0.87	1.90 ± 0.66
	Hypnone	-	-	-	2.32 ± 0.52	-	-	-
Acids/esters	Acetic acid	0.31 ± 0.16	0.52 ± 0.24	1.42 ± 0.12	2.53 ± 0.73	1.34 ± 0.41	2.88 ± 0.59	2.13 ± 0.81
	Propionic acid	-	-	0.79 ± 0.42	1.33 ± 0.39	3.31 ± 0.37	2.78 ± 0.56	3.54 ± 1.17
	Ethyl oenanthate	3.87 ± 0.82	3.52 ± 1.15	-	1.73 ± 0.21			
	Ethyl caprylate	-	-	0.91 ± 0.27	-	-	0.48 ± 0.19	-
	Ethyl acetate	-	2.75 ± 0.22	-	0.71 ± 0.12	0.85 ± 0.34	0.41 ± 0.15	0.66 ± 0.26
	Ethyl valerate	-	-	-	-	0.71 ± 0.23	0.96 ± 0.41	1.48 ± 0.36
Nitrogenous compounds	Hexylamine	0.89 ± 0.31	-	0.46 ± 0.18	-	-	-	-
	Ethanediamine	1.43 ± 0.35	-	-	1.09 ± 0.12	1.92 ± 0.45	-	-
	Trimethylamine	-	0.77 ± 0.26	2.80 ± 0.70	3.32 ± 1.02	3.67 ± 0.89	6.23 ± 2.40	7.59 ± 2.37
	Dimethylamine	-	-	-	1.26 ± 0.39	-	2.32 ± 0.94	-
	Heptylamine	-	-	-	-	0.80 ± 0.28	0.29 ± 0.09	-
Others	Carbon dioxide	11.20 ± 3.34	5.11 ± 1.72	2.32 ± 0.25	1.58 ± 0.31	3.28 ± 0.78	8.33 ± 2.04	15.23 ± 2.11
	Butylated hydroxytoluene	-	-	-	-	-	1.31 ± 0.22	3.45 ± 0.67

**Table 3:** The *R*, *G*, *B* values of fluorescent light photos of the SiQD-AgNC/PVDF film during beef spoilage.

Storage Time (d)	TVC of Beef (lg(CFU/g))	Color Parameters of SiQD-AgNC/PVDF Film			
		*R*	*G*	*B*	∆*C*
0	3.21 ± 0.33	156 ± 2.6	76 ± 1.3	220 ± 1.3	0
1	3.43 ± 0.24	150 ± 4.4	79 ± 2.5	221 ± 3.5	46
2	3.81 ± 0.35	140 ± 5.1	85 ± 3.3	225 ± 4.4	362
3	4.09 ± 0.31	128 ± 2.2	94 ± 4.7	229 ± 2.0	1189
4	5.41 ± 0.28	110 ± 3.3	109 ± 2.8	243 ± 0.9	3734
5	6.81 ± 0.26	96 ± 4.5	130 ± 3.1	255 ± 2.8	7741
6	7.65 ± 0.31	87 ± 3.5	150 ± 2.6	270 ± 1.9	12,737

## Data Availability

The data presented in this study are available on request from the corresponding author. The data are not publicly available due to product development privacy.
